# Does transfusion of residual cardiopulmonary bypass circuit blood increase postoperative bleeding? A prospective randomized study in patients undergoing on pump cardiopulmonary bypass

**DOI:** 10.4103/0973-6247.42659

**Published:** 2008-07

**Authors:** Rajnish Duara, Manoranjan Misra, Ritwick Raj Bhuyan, P. Sankara Sarma, Karunakaran Jayakumar

**Affiliations:** *Department of Cardiovascular and Thoracic Surgery, Achutha Menon Centre for Health Science Studies, Sree Chitra Tirunal Institute for Medical Sciences and Technology (SCTIMST), Thiruvananthapuram, Kerala-695 011, India*; 1 *Department of Biostatistics, Achutha Menon Centre for Health Science Studies, Sree Chitra Tirunal Institute for Medical Sciences and Technology (SCTIMST), Thiruvananthapuram, Kerala-695 011, India*

**Keywords:** Cardiopulmonary bypass circuit blood, residual pump blood, homologous blood transfusion, transfusion

## Abstract

**Objective:**

Homologous blood transfusion after open heart surgery puts a tremendous load on the blood banks. This prospective randomized study evaluates the efficacy of infusing back residual cardiopulmonary bypass (CPB) circuit i.e., pump blood as a means to reduce homologous transfusion after coronary artery bypass surgery (CABG) and whether its use increases postoperative drainage.

**Materials and Methods:**

Sixty-seven consecutive patients who underwent elective CABGs under CPB were randomized into 2 groups: (1) cases where residual pump blood was used and (2) controls where residual pump blood was not used. Patients were monitored for hourly drainage on the day of surgery and the 1^st^ postoperative day and the requirements of homologous blood and its products. Data were matched regarding change in Hemoglobin, Packed Cell Volume and coagulation parameters till 1st postoperative day. All cases were followed up for three years.

**Results:**

There was a marginal reduction in bleeding pattern in the early postoperative period in the cases compared to controls. The requirement of homologous blood and its products were also reduced in the cases.

**Conclusions:**

The use of CPB circuit blood is safe in the immediate postoperative period. The requirement of homologous blood transfusion can come down if strict transfusion criteria are maintained.

## Introduction

The world has seen a phenomenal increase in the number of open-heart surgeries done in cardiac centers in the last decade. The only constraint in the accelerating numbers has been the overwhelming demands for blood and blood products. Right from inception, cardiac surgery has been giving tremendous load on the blood banks to meet its ever-increasing demands. The risk of transmission of diseases like Hepatitis and HIV AIDS as well as transfusion related non-cardiogenic pulmonary edema and graft versus host disease has limited the use of blood and its products.[1] To counter these problems many new and indigenous blood conservation techniques like preoperative autologous blood collection to transfusion of shed mediastinal blood and strategies to redefine transfusion criteria have come up.[2] The last concept is very important as it works at the genesis of requirement, but recent surveys have shown the wide disparity amongst different centers regarding the threshold for blood transfusion.[1] Concern for excessive blood transfusion in cardiac surgery and the ever-increasing demand on the blood bank has been there from the very beginning. Way back in 1974, Zubiate *et al*, had given importance to conservation of blood in coronary artery surgery by standardizing a simplified technique with use of little blood in their early series of 477 patients.[3] We have used a known but forgotten technique to reduce the incidence of banked blood transfusion by collecting the residual blood in the cardiopulmonary bypass (CPB) circuit i.e., in the venous reservoir, the tubings and oxygenator in sterile blood bags and then transfusing it in the intensive care unit (ICU) in the immediate postoperative period.[2] There was concern on the part of the intensivist and the surgeon to transfusion of heparinised residual pump blood (RPB) to the patient due to increased chance of postoperative bleeding, as seen in some clinical observations in our institute in the recent past after use of RPB. Gravlee's study on heparin content of washed RBCs from the CPB circuit yielded heparin concentrations ranging from 0.08-0.22 USP U/ml and proved that it does not require supplemental protamine dose.[4] This prospective study was done to find out the feasibility of heparinised pump blood transfusion in terms of postoperative bleeding, safety in terms of clinical inflammatory signs and reduction in use of homologous blood and blood products.

## Materials and Methods

### Patients and study design

Sixty-seven consecutive patients who underwent coronary artery bypass grafting (CABG) during a three-month period from September 2004 to December 2004 were included in this prospective study. All the patients were followed-up for three years after completion of the study. The patients were divided into two groups, cases (n=33, 5 females) where residual CPB circuit (pump) blood (RPB) was used and controls (n=34, 4 females) where residual pump blood was not transfused. The RPB was transfused by the intensivist in the immediate postoperative period in the intensive care unit within six hours of shifting from the theatre. Patients with only venous grafts, emergency CABGs, CABG with other procedures, abnormal coagulation parameters, continuing heparin therapy and deranged liver function tests were excluded from the study. Inclusion criteria were all elective cases and use of left internal mammary artery (LIMA) as a conduit. Aspirin and clopidrogel were stopped one week prior to surgery in all patients.

Oxygenated pump blood was collected by the perfusionist under supervision of the anesthesiologist in sterile empty blood bags under aseptic and sterile conditions from the arterial line port of the CPB circuit after one-third protamine dose and removal of the arterial cannula. Surgeon and anesthesiologists were blinded as to the group of randomization until the patient was received in the ICU as RPB was collected by the perfusionist. No aprotinin or extra protamine and hemostatic agents were used in both the groups. Aspirin and clopidrogel were started on the first postoperative day routinely. Patients who underwent endarterectomies were started on low molecular dextran (20%) in saline @ 20cc/h after six hours of shifting to the ICU for 48h.

### Operative procedure

All patients underwent on-pump CABG by a single surgeon (JK) under primary median sternotomy and routine CPB with moderate hypothermia (28 degrees Celsius) and tepid antegrade blood cardioplegia was used. A pedicled LIMA to left anterior descending artery and great saphenous vein to other targets were grafted in all cases.

### Anaesthetic technique

The anesthesia consisted of morphine and diazepam for induction, nitrous oxide and isoflurane for maintenance and pancuronium for muscle relaxation.

### Cardiopulmonary bypass

Crystalloid priming of CPB circuit was with 1000ml of Ringer's lactate. Heparinisation was done @3 mg/kg with achievement of ACT of more than 450 sec. Cardiopulmonary bypass was instituted with aorto – two stage right atrial cannula using roller pump and membrane oxygenator (Capiox E, Terumo Corporation, Tokyo Japan). All patients were cooled to 28 degree Celsius. Hematocrit was kept between 22-26%. Heparin was fully neutralized with protamine at 1:1 ratio. No extra protamine was given after shifting the patient to the ICU.

### Collection of CPB circuit blood

The residual blood was collected by the perfusionist in sterile blood bags at the end of the procedure after one-third protamine neutralization and transfused by the intensivist within the next six hours in the ICU.

### Blood sampling

Hemoglobin and PCV estimation of the RPB was done in all cases. Each group was subjected to hemoglobin, PCV, platelet counts, INR tests on day of surgery and first postoperative day.

### Transfusion policies

The transfusion criteria were set in terms of full replacement in terms of drainage on day of surgery to limit biasness as a part of study protocol.

### Outcome measures

The cases and controls were matched according to the preoperative data like age, sex, preoperative hemoglobin, packed cell volume (PCV), international normalized ratio (INR) of prothombin time (PT) and platelet count. Postoperatively both data were matched in terms of total heparin and protamine dose, number of conduits, duration of surgery, CPB and aortic cross clamp times. Both groups were compared in relation to mediastinal drainage in the first six hours, 12 hours, 24 hours and total drainage. Both groups were assessed in terms of amount of use of homologous blood and blood products, ICU and hospital stay. The safety of use was determined by presence or absence of clinical features of inflammation like fever and post pericardiotomy syndrome. All patients were followed-up for three years after surgery.

### Statistical analysis

All statistical evaluation was done using Microsoft^®^ SPSS software, version 10 (SPSS, Inc., Chicago, IL). Descriptive statistics for the groups are presented as mean ± standard deviation or as a simple percentage. Differences within and between groups were analyzed using the two-tailed unpaired or paired Student's t-test or Fischer's exact test as appropriate, when continuous and dichotomized variables were compared. The Mann-Whitney U test was used to compare postoperative blood loss and transfusion requirements, which do not follow a Gaussian distribution. A *P* value of <0.05 was considered statistically significant.

## Results

Sixty-seven patients were randomized out of the entire elective CABGs performed between September 2004 and December 2004. Thirty four cases (controls) where RPB was avoided and rest of the 33 patients (cases) had RPB transfused in the immediate postoperative period in the ICU. Both the groups were matched according to demography and preoperative investigations like Hemoglobin, PCV, INR and platelet counts. The mean ages, 54.6 ± 8.2 years in controls and 56.4 ± 9.3 years in cases, did not differ significantly (*P*=0.410). Mean preoperative Hemoglobin, 14.2 ± 1.3 gm% in controls and in cases 14.1 ± 1.6 gm% (*P*=0.717); mean INR 1.23 ± 0.12 in controls and 1.27 ± 0.17 in cases (*P*=0.319) and mean platelet count 2.3 ± 0.5 in controls and 2.4 ± 0.5 in cases (*P*=0.608) were not significantly different. The operative data in both groups as shown in Table 1 is not statistically significant.

**Table 1:** Operative data showing comparable variables between the two groups

Operative data	Controls	Cases	*P* value
No. of grafts	3.88 ± 0.81	3.85 ± 0.97	0.877
Patients with endarterectomy	9 (26.5%)	3 (9.1%)	0.065
Total surgery time (min)	209.4 ± 29.3	202.9 ± 37.2	0.427
CPB time (min)	92.8 ± 17.6	95.7 ± 28.3	0.618
Cross clamp time (min)	54.9 ± 10.1	54.1 ± 14.6	0.778
Total heparin dose (mg)	245.2 ± 41.1	265.8 ± 72.6	0.156
Total protamine dose (mg)	267 ± 52	274±58.7	0.575

**Figure 1:**
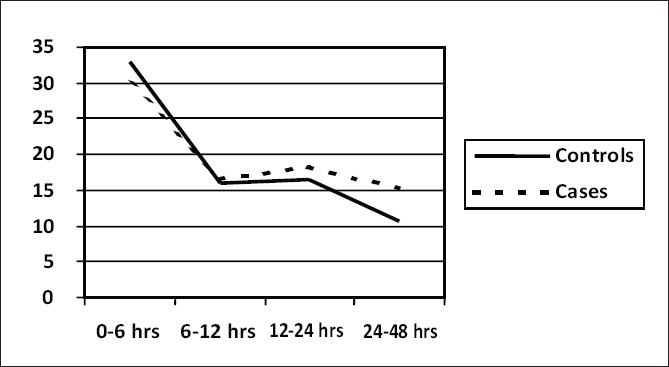
Line diagram showing bleeding pattern in controls and cases

The postoperative bleeding data is shown graphically in Figure 1. The mean total drainage in controls were 701.47 ± 304.54 ml and 666.67 ± 325.53 ml in cases. Although this was not statistically significant, drainage is marginally low in the group where RPB was used. The RPB hemoglobin was 8.66 ± 1.56 gm/dl in cases and 8.56 ± 1.57 gm/dl in controls. Mean of 345. 2 ± 156.7 ml of RPB was used in the study group. 305.6 ± 245.2 ml and 245.8 ± 236.4 ml of packed RBCs were used in controls and cases respectively. The postoperative hemoglobin, PCV and platelet counts in both controls and cases were similar [Table 2]. The ICU stay was in the range of 36 to 72h in controls (mean of 46h) and 36 to 68h in cases (mean of 45 h). Hospital stay was same in both controls and cases with mean of 5days (Min 5days; Max 7days). All the patients in both groups were followed up for 3 years and were in NYHA functional class I.

**Table 2:** Postoperative data

Postoperative data	Controls	Cases
Day 0 Hb (gm/dl)	11.66 ± 1.33	11.56 ± 1.77
Day 1 Hb (gm/dl)	11.99 ± 1.24	12.01 ± 1.59
Day 0 PCV (%)	37.6 ± 3.9	37.2 ± 5.3
Day 1 PCV (%)	36.6 ± 2.9	37.2 ± 3.9
Day 0 Platelet count (no/dl)	1.83 ± 0.72	1.76 ± 0.46
Day 1 Platelet count (no/dl)	1.73 ± 0.49	1.57 ± 0.39
Day 0 INR	1.47 ± 0.22	1.48 ± 0.13
Day 1 INR	1.44 ± 0.17	1.51 ± 0.22

## Discussion

Conservative blood transfusion is the norm in modern cardiac surgical practice.[1,2] Post surgical patients can tolerate normovolemic hemodilution after myocardial revascularisation as evidenced by absence of any correlation between hematocrit values and exercise capacity in the early postoperative period.[5] Jones *et al*, had used profound hemodilution during CPB with added complementary conservation measures to reduce homologous transfusion.[6] But an earlier study has cautioned against potential cardiac injury with hemoglobin of less than 7 gm/dl in postoperative cardiac surgical patients.[7] In spite of these, there had been a lack of strict transfusion protocol after cardiac surgery and these are variable in different centers, pertaining to the whims and fancies of health care providers.[1,8] The risk of transfusion related complications far outweigh the beneficial effects of homologous transfusion. The strategies required to achieve this had been traditionally the following: (1) predonation of autologous blood for elective surgery, (2) intraoperative withdrawal of autologous blood, (3) normovolemic hemodilution using crystalloid prime, (4) intraoperative use of cell savers, (5) intraoperative hemofiltration, (6) postoperative autotransfusion of shed mediastinal blood and (7) pharmacologic interventions (e.g., desmopressin acetate, aprotinin, epsilon aminocaproic acid).[9,10] All these methods require either equipment or resources; predeposition requires skilled people to collect blood timely and keep it ready for subsequent surgery; cell savers and hemofilters add to the cost; autotransfusion of shed mediastinal blood requires a special device and maintenance of sterility is a must; pharmacologic agents are double edged swords and not too many coronary surgeons are happy to use aprotinin after revascularisation. The present study was undertaken to find out the clinical safety of use of RPB in terms of postoperative bleeding and efficacy in terms of reducing banked blood transfusions in CABG cases. Most of the time the amount left in the CPB circuit is substantial and rather than discarding it, has tremendous potential in transfusing in the immediate postoperative period in the ICU. CABG cases are a good study group as it has higher incidence of bleeding compared to valvular and pediatric cardiac surgery. We have collected RPB in sterile empty blood bags and used as initial maintenance fluid @ 2-4ml/kg depending on the requirement within 6 hours of shifting from the theatre. The method used is simple and does not add to the cost of usual transfusion. Earlier studies have shown very minimal heparin content of RPB.[4] We have also not given any extra protamine to counter residual heparin in the pump blood transfused. The benefit of autologous transfusion done by withdrawal of blood prior to CPB from peripheral or central veins, arterial line or from extracorporeal tubings and storing either in acid-citrate-dextrose, citrate-phosphate-dextrose or heparinised solutions at room temperature and reinfusing after CPB into a peripheral vein or heart has long been well accepted.[8] But this also has some limitations in CABGs like low hemoglobin especially in third world countries where anemia is still a major issue. Pre CPB autologous blood letting may cause hemodynamic instability in patients with left main disease and severe triple vessel disease due to relative ischemia. Another variant of autologous transfusion is transfusion of shed mediastinal blood. Numerous studies have shown its safety and efficacy in lowering homologous transfusion requirements.[10–13]

Schaff *et al*, used a mediastinal drain collection unit which had two170 micron screens and placed under–20 cm H_2_ O pressure and could reduce bank blood requirements by 50%.[11] But it had limitations as 6 units grew positive cultures though majority were skin and air contaminants and the amount of free plasma hemoglobin was more than six times higher than banked blood. Kochamba *et al*, showed 28% reduction of chest tube drainage in first eight hours and 45% reduction in total homologous blood units transfused.[12] Chest tube drainage fluid was washed and centrifuged before transfusing in the study by Dalrymple-Hay had showed its benefit by reducing homologous transfusions.[14] There had been doubts regarding the efficacy of the shed mediastinal blood especially on its survival time and oxygen transport capacity. Schimdt *et al*, showed no changes in the 2, 3–diphosphoglycerate (2,3-DPG) concentration, calculated half saturation tension (p50) and concentration of extractable oxygen (Cx) between shed mediastinal blood and patient-blood.[15,16] They also labeled shed mediastinal RBCs and normal circulating RBCs with 99mTc and 51Cr and demonstrated similar survival times.[17] However, a prospective randomized trial of autotransfusion of shed mediastinal blood after routine cardiac operations showed increase in requirement of homologous blood transfusion and also had elevation of fibrin split products in the autologous group.[18] Ferraris *et al*, showed the futility of using autologous blood reinfusion methods in low risk patients determined by ratio of preoperative bleeding time to preoperative RBC volume.[10] Tyson *et al*, highlighted the merits of multi-technique approach of blood conservation and stressed that "commitment" to blood conservation is more important than any particular technique.[19] In one of the largest prospective series on autotransfusion of shed mediastinal blood from Mayo Clinic the authors showed increased bleeding in the first 24h period after transfusing shed mediastinal blood (1721±1510 ml versus 1278 ± 814 ml in non autotransfused group); the use of banked blood in the autotransfused group was 39% compared to 56% in the nonuse group.[20] Looking into all these perspectives it seems that use of shed mediastinal blood requires a sterile collecting device and the complexity of collecting and retransfusing it. RPB is already present in the circuit and only need to be collected at a single time in the theatre from the arterial line of the CPB circuit. It does not require any anticoagulation as it is already heparinised with just one-third dose of protamine after stopping the pump and all the suction catheters. The present study shows the use of RPB does not produce any increased bleeding and drainage is less than the control group (666.67 ± 325.53 ml compared to 701.47 ± 304.54 ml in controls).

The use of banked blood can come down further if strict transfusion criteria are maintained. There is a risk of exaggerated systemic inflammatory response to use of RPB containing all the systemic inflammatory mediators and increased leukocyte counts, but the present study do not show any evidence of systemic response to use of RPB as evidenced by pyrexia or pericardial effusion. The beneficial effects of leukocyte free blood transfusion are well-documented.[21,22] A recent study from Netherlands compared transfusion of leukocyte freed RPB from nonfiltered RPB; although the sample size was small, the study concluded that circulating leucocytes were significantly diminished with the filtered group and could reduce the postoperative inflammatory response in patients undergoing cardiac surgery.[23] Use of RPB is safe, simple, and cost effective and reduces the load on the blood bank. Subject to strict transfusion protocol, RPB use can definitely reduce multiple transfusions and in effect reduce the transfusion related complications. Given at a maintenance fluid infusion rate, it does not increase the mediastinal drainage as had been evident from our study. Also, it did not require any extra protamine dosage to counter the minimal residual heparin content of the pump blood as it was shown by Gravlee's study.[4] This study is unique as it is the only prospective randomized study to deal with use of RPB in CABG subsets in terms of determining the transfusion load and amount of mediastinal drainage. The study has some limitations, as it is a single institutional study with a small cohort size. It also lacks quantitative data on the inflammatory response markers after use of the CPB circuit blood. The RPB was administered without removing leucocytes and its efficacy in comparison to leukocyte free RPB requires further evaluation. But overall it has shown its clinical efficacy in reducing transfusion of homologous-banked blood without any concomitant rise in mediastinal drainage after its use. We recommend use of residual pump blood in the immediate postoperative period in the ICU after all open heart surgeries.
